# Is High Resolution Melting Analysis (HRMA) Accurate for Detection of Human Disease-Associated Mutations? A Meta Analysis

**DOI:** 10.1371/journal.pone.0028078

**Published:** 2011-12-14

**Authors:** Bing-Sheng Li, Xin-Ying Wang, Feng-Li Ma, Bo Jiang, Xiao-Xiao Song, An-Gao Xu

**Affiliations:** 1 Department of Gastroenterology, Nanfang Hospital, Southern Medical University, Guangzhou, People's Republic of China; 2 Guangdong Provincial Key Laboratory of Gastroenterology, Guangzhou, People's Republic of China; 3 School of Public Health, Kunming Medical University, Kunming, People's Republic of China; 4 Huizhou Medicine Institute, Huizhou First Hospital, Huizhou, Guangdong, People's Republic of China; University of Vermont, United States of America

## Abstract

**Background:**

High Resolution Melting Analysis (HRMA) is becoming the preferred method for mutation detection. However, its accuracy in the individual clinical diagnostic setting is variable. To assess the diagnostic accuracy of HRMA for human mutations in comparison to DNA sequencing in different routine clinical settings, we have conducted a meta-analysis of published reports.

**Methodology/Principal Findings:**

Out of 195 publications obtained from the initial search criteria, thirty-four studies assessing the accuracy of HRMA were included in the meta-analysis. We found that HRMA was a highly sensitive test for detecting disease-associated mutations in humans. Overall, the summary sensitivity was 97.5% (95% confidence interval (CI): 96.8–98.5; I^2^ = 27.0%). Subgroup analysis showed even higher sensitivity for non-HR-1 instruments (sensitivity 98.7% (95%CI: 97.7–99.3; I^2^ = 0.0%)) and an eligible sample size subgroup (sensitivity 99.3% (95%CI: 98.1–99.8; I^2^ = 0.0%)). HRMA specificity showed considerable heterogeneity between studies. Sensitivity of the techniques was influenced by sample size and instrument type but by not sample source or dye type.

**Conclusions/Significance:**

These findings show that HRMA is a highly sensitive, simple and low-cost test to detect human disease-associated mutations, especially for samples with mutations of low incidence. The burden on DNA sequencing could be significantly reduced by the implementation of HRMA, but it should be recognized that its sensitivity varies according to the number of samples with/without mutations, and positive results require DNA sequencing for confirmation.

## Introduction

Although DNA sequencing, including direct DNA sequencing and pyrosequencing [1], is considered as the “gold standard” for known/unknown mutation scanning, it still remains relatively expensive, laborious and time-consuming. Many other methods for mutation scanning have been developed to screen for differences between the two copies of DNA within an individual. These techniques include single-strand conformational polymorphism analysis (SSCP) [2], denaturing gradient gel electrophoresis (DGGE) [3], denaturing high performance liquid chromatography (DHPLC) [4], temperature gradient capillary electrophoresis (TGCE) [5] and mass spectroscopy [6]. All of these methods require separation of the sample on a gel or other matrix. Fluorescence monitoring of PCR product melting profiles is another alternative to DNA sequencing that permits the detection of DNA mutations in solution without the need for separation on a gel or other matrix [7]. Fluorescently labeled, probe-based methods, such dual hybridization [8], exonuclease (TaqMan) [9], or hairpin (Molecular Beacon) [10] probes, may be used for mutation detection, but only for the bases covered by the probe. Hence, these methods are not amenable to mutational scanning as mutational scanning requires methods that can detect mutations over larger regions. Furthermore, some of the above methods are not automated and are therefore labor intensive while others are complex, costly and require specialized instrumentation.

High resolution melting analysis (HRMA) is a simple, PCR-based method. In the presence of saturating concentrations of DNA binding dyes, the specific sequence of the amplicon determines the melting behavior as the temperature of the solution is increased. Fluorescence intensity decreases as the double stranded DNA becomes single stranded and the dye is released. The melting temperature (Tm) at which 50% of the DNA is in the double stranded state can be approximated by taking the derivative of the melting curve. The distinctive melting curve can used to detect DNA sequence variations in the amplicon without the need for any post-PCR processing. The method is easy to use, highly sensitive, specific, low cost and yields rapid sample turn-around [11]–[13], making HRMA an attractive choice for the detection of disease-associated mutational variants with applications in clinical diagnostic labs. Furthermore, HRMA is a nondestructive method. Therefore, subsequent analysis of the sample by other techniques, such as gel-electrophoresis or DNA sequencing, can still be performed after HRMA analysis. These characteristics make HRMA ideal for use in routine diagnostic settings. Due to its numerous advantages, HRMA has been widely applied in diagnostic laboratories for screening for disease-associated mutations. Since it was first introduced for genotyping in 2003 [14], HRMA has been used to detect mutations such as EGFR [15], [16], KRAS [13], [17], KIT [18], BRAF [19], [20], BRCA [21], TP53 [22]. In the setting of the EuroGenTest consortium, inter-laboratory evaluation and validation of HRMA, and generation of guidelines for implementing the method as a scanning technique for the discovery of new genes have been proposed [21]. One disadvantage of HRMA is that the sensitivity and specificity in an individual clinical diagnostic setting are variable [23]. According to the “OECD Guidelines for Quality Assurance in Molecular Genetic Testing” [24], there is an obligation for diagnostic laboratories to provide high quality results. Therefore, all methods implemented within a routine setting must be duly validated and achieve acceptable limits for sensitivity and specificity prior to their diagnostic use. Although reviews and reports on the use of HRMA for mutation scanning and genotyping have been published previously [23], [25]–[29], a systematic review of the application of the technique for diagnostic purposes has not been carried out. Therefore, the meta-analysis described in this study was performed to evaluate the diagnostic accuracy of HRMA and investigate the potential for implementation of HRMA in different routine clinical settings for the detection of human disease-associated mutations. The analysis includes a comparison to DNA sequencing. The purpose of the analysis is to provide clinicians and health managers with a more objective basis for decision-making regarding implementation of the technique and to assess areas where there is currently a lack of evidence regarding the technique [30].

## Materials and Methods

### Literature search strategy

A literature search was carried out between July and November 2010 using the following databases: Medline, Embase, Cochrane Library and the Medion databases. The following search words (all fields) were used: ‘high resolution melting analysis or high-resolution fluorescent melting curve analyses or High-resolution amplicon melting analysis’, ‘HRM or HRMA or HRMCA’, ‘mutation’, and ‘sequence or sequencing’. The CBMdisc databases were used for Chinese articles with the following keywords (in Chinese): ‘HRM or HRMA or HRMCA’ and ‘sequencing’. The results were limited to human species. The date of publication was limited to November 6, 2010. In addition, the following journals were screened manually: Human Mutation, Cancer Research, Human Molecular Genetics, Clinical Chemistry, Genetic Testing, Clinical Genetics, Nucleic Acid Research and the Journal of Medical Genetics and Human Genetics. Furthermore, the reference lists of the included studies were screened and additional search engines, including SUMsearch, TRIP database, Sciencedirect, Google, Database for Chinese Journals of Technology (Chinese) were used. The applicability of borderline publications was discussed by the authors until a consensus for inclusion or exclusion was reached. The Institutional Review Boards approved the conclusion that no ethical approval was required for this study.

### Inclusion and exclusion criteria

The inclusion criteria were as follows: (1) HRMA was applied to the study of disease-associated mutations in humans, (2) sequencing (including direct sequencing, dideoxy sequencing or sequencing of HRM products) was used as a reference standard, (3) only parts of mutated genes were investigated, (4) only some study data was compared to direct sequencing as a reference standard (only this data was included in the current study), (5) sensitivity and specificity were reported or could be calculated from the results reported, (6) the authors only reported that there were no false positive or false negative results, so that conclusions on sensitivity and specificity could be drawn without calculation of these parameters, (7) all fragments were included if one gene locus was amplified into multiple fragments and (8) the publication language was English or Chinese. The exclusion criteria were as follows: (1) studies were performed using only HRMA or comparing HRMA with non-sequencing techniques, (2) HRMA was combined with other detection methods, such as probes or qPCR, (3) studies used samples with artificially created sequences, (4) studies were aimed at detecting polymorphisms. Non-systematic/narrative reviews, letters, comments, and meeting abstracts were also excluded. Unpublished sources of data were not included. Publications identified as duplicates were excluded.

### Assessment of Study Quality

The quality of the studies was assessed according to the “Quality Assessment of Diagnostic Accuracy Studies” (QUADAS) tool [31]. The modified tool was composed of 10 item questions summarized in Table 1, which were each answered “yes,” “unclear,” or “no.” Quality assessment of the studies was carried out independently by two reviewers (B.S. Li and F.L. Ma). If the quality assessment of the two reviewers were not in agreement, the discrepancies were resolved by consensus. The tool does not incorporate a global quality score. The main reason for this is that quality scores ignore the importance of individual items and potential biases related to individual items may vary according to context. Therefore, the application of quality scores may dilute or ignore potential associations [31].

**Table 1:** “Quality Assessment of Diagnostic Accuracy Studies” (QUADAS) Tool.

Author	1	2	3	6	8	9	10	11	13	14
Bastien, R. (2008)	N	Y	Y	Y	Y	Y	Y	Y	Y	Y
Dagar, V. (2009)	N	Y	Y	Y	Y	U	U	U	Y	Y
Do, H. (2008)	Y	Y	Y	Y	Y	Y	Y	Y	Y	Y
Doi, Y. (2009)	N	Y	Y	Y	Y	N	U	U	Y	Y
Fassina, A. (2009)	Y	Y	Y	Y	U	U	U	U	Y	Y
Franklin, W.A. (2010)	N	Y	Y	Y	Y	Y	U	U	Y	Y
Fukui, T. (2008)	N	Y	Y	N§	U	U	U	U	Y	Y
Fuster, O. (2009)	N	Y	Y	Y	Y	Y	U	U	Y	Y
Gaucher, C. (2009)	N	Y	Y	Y	Y	U	U	U	Y	Y
Hung, C.C. (2008)	N	Y	Y	Y	Y	U	N	Y	Y	Y
Krenkova, P. (2009)	N	Y	Y	Y	Y	N	U	Y	Y	Y
Krypuy, M. (2006)	N	Y	Y	Y	Y	Y	U	U	Y	Y
Krypuy, M. FF (2007)	N	Y	Y	Y	Y	Y	Y	Y	Y	Y
Krypuy, M. FFPE (2007)	N	Y	Y	Y	Y	Y	U	U	Y	Y
Liyanage, K.E. (2008)	N	Y	Y	Y	Y	Y	Y	Y	Y	Y
Lopez-Villar, I. (2010)	N	Y	Y	Y	Y	Y	U	U	Y	Y
Ma, E.S. (2009)	N	Y	Y	Y	Y	Y	N	Y	Y	Y
Nomoto, K. (2006)	N	Y	Y	Y	Y	Y	U	Y	Y	Y
Olsen, R.K. (2010)	N	Y	Y	Y	Y	Y	Y	Y	Y	Y
Pichler, M. (2009)	N	Y	Y	Y	Y	Y	Y	Y	Y	Y
Polakova, K.M. (2008)	N	Y	Y	Y	Y	Y	U	U	Y	Y
Rapado, I. (2009)	Y	Y	Y	Y	Y	Y	U	U	Y	Y
Simi, L. (2008)	N	Y	Y	Y	Y	Y	U	U	Y	Y
Takano, T. (2007)	N	Y	Y	Y	U	U	Y	U	Y	Y
Tan, A.Y. (2008)	N	Y	Y	Y	Y	Y	U	U	Y	Y
van Eijk, R. (2010)	N	Y	Y	Y	Y	Y	U	U	Y	Y
Whitehall, V. (2009)	N	Y	Y	Y	Y	Y	U	U	Y	Y
Willmore, C. (2004)	N	Y	Y	Y	Y	N	U	U	Y	Y
Willmore-Payne, C. (2005)	N	Y	Y	Y	Y	Y	U	Y	Y	Y
Willmore-Payne, C. (2006 LC)	N	Y	Y	Y	Y	N	U	Y	Y	Y
Willmore-Payne, C. (2006)	N	Y	Y	Y	Y	N	U	Y	Y	Y
Xiao, J. (2009)	N	Y	Y	Y	Y	U	U	U	Y	Y
XinHui,Fu. (2009)	N	Y	Y	Y	Y	Y	U	U	Y	Y
YongPing,Lu. (2010)	N	Y	Y	Y	Y	U	U	U	Y	Y
ZhiHong,Chen. (2010)	N	Y	Y	Y	Y	Y	U	U	Y	Y

Y = yes; N = no; U = unclear;

§ reference standard included sequencing and pyrosequencing; LC: lung cancer.

Items: 1) Was the spectrum of patients representative of the patients who will receive the test in practice? 2) Were the selection criteria clearly described? 3) Is the reference standard likely to classify the target condition correctly? 6) Did patients receive the same reference standard regardless of the index test result? 8) Was the execution of the index test described in sufficient detail to permit replication of the test? 9) Was the execution of the reference standard described in sufficient detail to permit replication? 10) Were the index test results interpreted without knowledge of the results of the reference standard result? 11) Were the reference standard results interpreted without knowledge of the results of the index test? 13) Were uninterpretable/intermediate test results reported? 14) Were withdrawals from the study explained?

### Outcome parameters

The outcome parameters were sensitivity, specificity, positive predictive value and negative predictive value. Two ‘statistical’ units (per amplicon and per sample) and different definitions of ‘positive result’ were accepted as a basis for the calculation of these parameters. For example, ‘positive’ could mean any alteration, such as mutations, undetermined melting curves and polymorphisms.

### Data extraction

The two reviewers (B.S. Li and F.L. Ma) independently extracted relevant data from each article using a standardized form (Table S1). The reviewers were not blinded with regard to information about the journal name, author names, author affiliations or year of publication since this has previously been shown to be unnecessary [32]. To resolve disagreement between reviewers, other authors assessed all discrepant items and the majority opinion was used for analysis.

### Study characteristics

The QUADAS quality assessment tool was used to extract the relevant study design characteristics of each study (Table 1). In addition, other main study characteristics were recorded as follows: (1) year of publication, (2) disease type, (3) sample source, (4) prevalence of samples with mutation, (5) target fragment/mutation-type analyzed, (6) instrument used (7) dye used, (8) level of analysis (per amplicon and per sample) and (9) length of sequence (Table 2). The following features were also extracted: (1) sample size, (2) study site, (3) language and (4) design type.

**Table 2:** Result of the multivariable meta-regression model for the most important characteristics with backward regression analysis (Inverse Variance Weights).

Variable	Coeff.	Std. Err.	p - value	RDOR	[95%CI]
**Cte.**	1.838	0.9233	0.0516	-	-
**S**	−0.629	0.1301	0.0000	-	-
**Instrument type**	0.440	0.2573	0.0930	1.55	(0.93; 2.60)
**Sample size**	2.577	0.5975	0.0001	13.15	(3.97; 43.57)

### Examination results

2×2 tables were extracted on per sample or per amplicon basis, including the numbers of true-positive, true-negative, false-positive, and false-negative results in the detection of disease-associated mutations (Table S1).

### Statistical analysis

Combined estimates of sensitivity, specificity, positive and negative likelihood ratios (LRs) and diagnostic odds ratio (DOR), together with their 95% confidence intervals (CI), were obtained from the available data reported in the selected studies (proportions of true positives, true negatives, false positives and false negatives). To handle studies with empty cells, 0.5 was added to all cells from all studies.

The heterogeneity of all indices was evaluated by graphical examination of forest plots, which are commonly used to detect heterogeneity in meta-analysis. As meta-analyses include small numbers of studies, the power of the usual Cochran's Q test is low. Therefore, they are poor at detecting true heterogeneity among studies as significant. An alternative approach to quantify the effect of heterogeneity is the I^2^ index that describes the percentage of total variation across studies that is due to heterogeneity rather than chance [33]. I^2^ is calculated and a value >50% indicates substantial heterogeneity [33]. Meta-analyses were performed by combining the sensitivities, specificities, LRs and DORs using the DerSimonian-Laird method, a random effects model [34], in order to incorporate variations among the studies. This approach was taken because including random effects has been previously reported as the more realistic and appropriate model for this type of meta-analysis [35], [36]. As a “threshold effect” was not detected by the Spearman test and the examination of sensitivity and specificity plots on a receiver operating characteristic (ROC) plane, summary receiver operating characteristic (SROC) curves were not constructed [37]. The analyses were carried out using Microsoft Excel 2003 (Microsoft, Seattle, WA, USA), SPSS 13.0 for Windows (SPSS, Chicago, IL, USA) and Meta-DiSc (Version 1.4) [38].

### Meta-regression analysis

Meta-regression analysis was executed to determine whether diagnostic values were significantly affected by heterogeneity between the individual studies. First, single factor regression analysis was performed using variates including instrument type (HR-1 or other instrument (LightCycler4 80, Rotor-Gene 6000, LightScanner 96)), level of analysis (per amplicon or per sample), dye type (EvaGreen, LCGreen I, LCGreen plus, Resolight or Syto9), sample source (blood cell/bone marrow, fresh frozen (FF) tissue, formalin-fixed and paraffin-embedded (FFPE) tissue or cytologic slides), eligible/non-eligible sample size (eligible (>35 samples/amplicons with mutations and >35 samples/amplicons without mutation to yield 95% confidence intervals whose lower boundary is >90% sensitivity if the sensitivity is 100%) [39] and non-eligible (all other samples), disease type (tumorous or non-tumorous), study site (Europe, Asia, Oceania or North America), mutation type (TP53, EGFR, KRAS or others), study language (Chinese or English), design type (single-gate design or two-gate design) and answers from the 10 questions of the QUADAS quality assessment tool. Variates were considered as explanatory if their regression coefficients were statistically significant (P<0.05). Subsequently, we developed a multivariable regression model and using a backward stepwise algorithm, we identified the most important characteristics. Characteristics were retained in the regression model if P<0.05.

### Subgroup analyses

Subgroup analyses were planned *a priori* depending on the following: (1) most important characteristics as selected via meta regression, (2) instrument type (HR-1 or other instrument), (3) dye type (EvaGreen, LCGreen I, LCGreen plus, Resolight or Syto9) and (4) sample source (blood/bone marrow cell, FF tissue, FFPE tissue or cytologic slides).

## Results

### Literature search outcome

**Figure 1:**
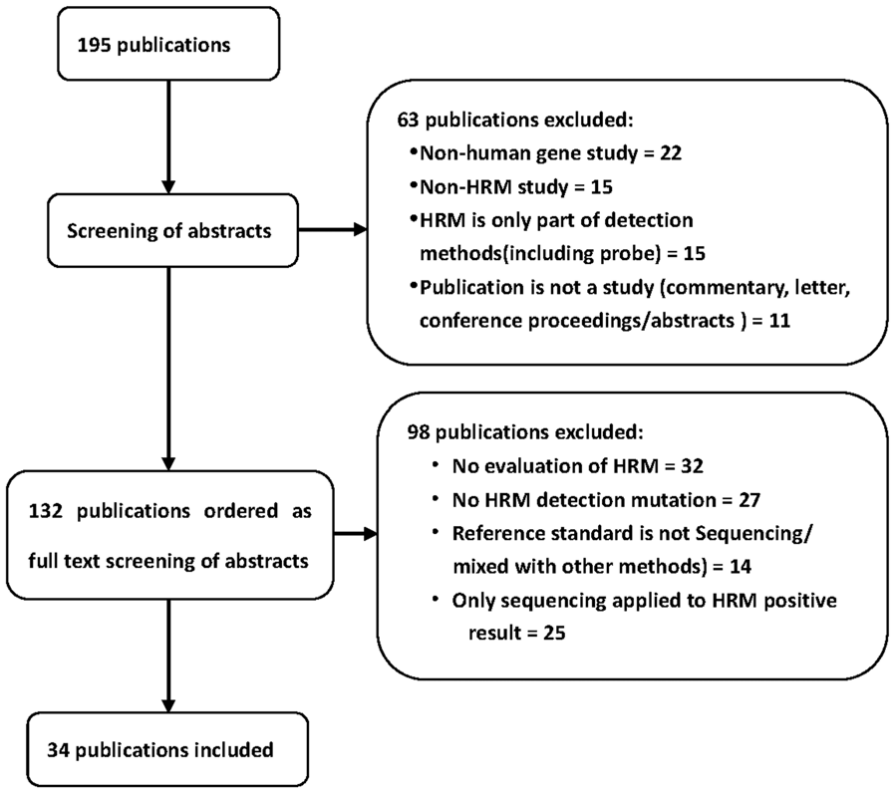
Flowchart for the selection of articles for meta-analysis.

The results of the literature search and the stepwise exclusion process are illustrated in Figure 1. Out of 195 references found, only 34 articles met our inclusion criteria. These articles were divided into 58 ‘units’ for statistical analysis according to target fragment/mutation-type and sample source (Table S1). Of the 161 publications excluded, 22 were for non-human HRMA studies, such as viruses, bacteria, mosquitoes and other animals, 15 were non-HRMA studies applied to the human genome, 15 were studies where HRMA was used as part of other research methods, seven used multiple probes, eight combined HRMA with qPCR or other methods, 11 were not original research studies, one was a conference presentations, eight were reviews, two were letters, 32 were not for performance evaluation studies of HRMA, 27 were not of HRMA applied to mutation detection (20 SNPs, 4 methylation and 3 others), 14 did not exclusively use sequencing as the reference standard (8 dHPLC, 1 DGGE and 5 mixed methods) and 25 only applied sequencing to HRMA positive results. Amplicon size varied from 51–634 bp and the most common sample source was FFPE. The most frequently used dye was LCGreen I and the most commonly used instrument was the LC480.

### Study description

**Figure 2:**
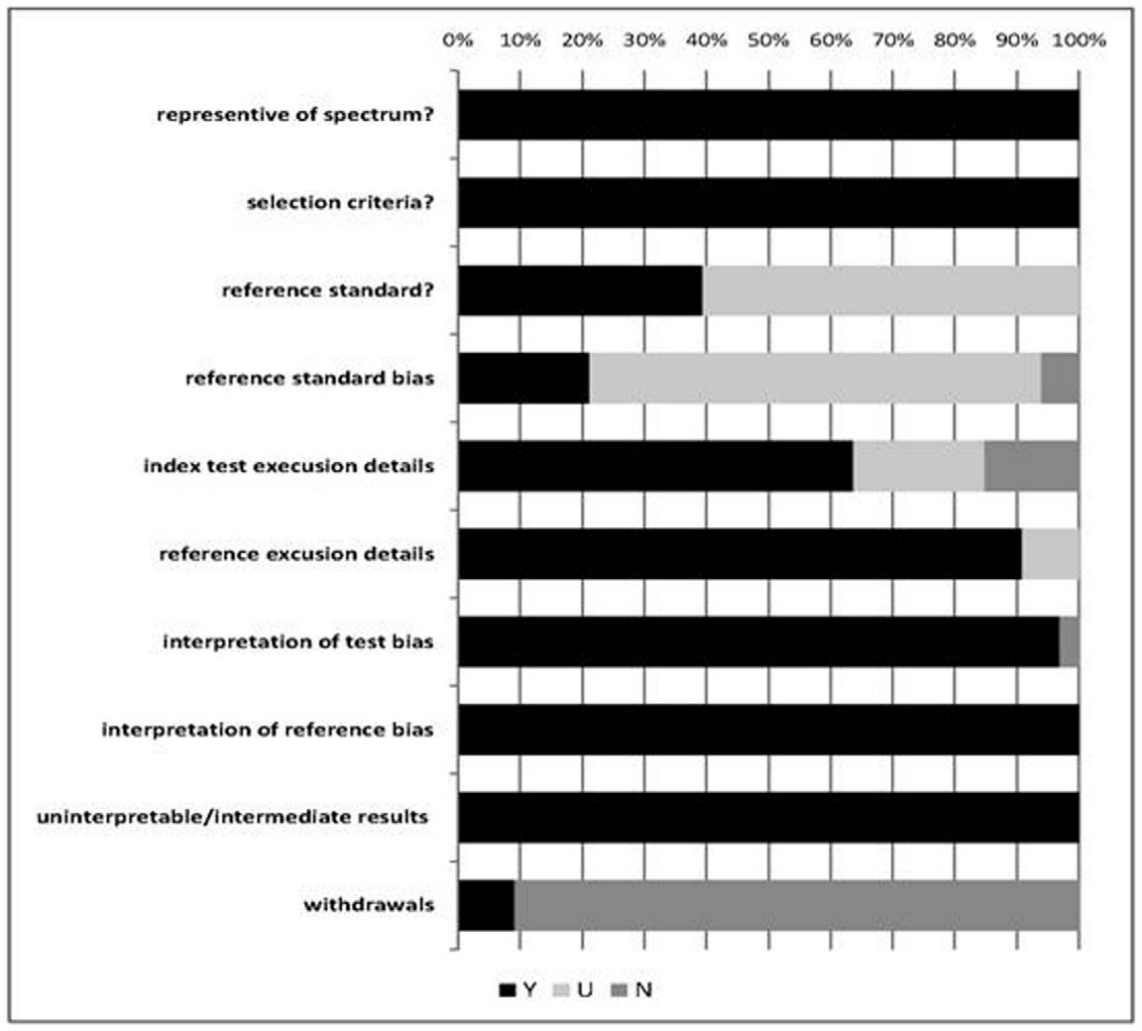
Assessment of quality items using modified QUADAS tool. Y = yes; U = unclear; N = no.

The 34 studies included in the meta-analysis included reports on the evaluation of the accuracy of HRMA for the detection of human disease-associated mutations (Table S1). Instrument types included HR-1 [15], [16], [19], [40]–[45] (n = 9), LightCycler480 [13], [17], [18], [20], [22], [46]–[55] (n = 14), RotorGene6000 [13], [56]–[62] (n = 8) and LightScanner96 [63]–[65] (n = 3). There were 27 single-gate designs and 7 two-gate designs. Disease types included 27 tumorous and seven non-tumorous diseases. The total answers to the QUADAS quality assessment tool included, ‘yes’ 251/350 (71.7%) and ‘unclear/no’99/350 (28.3%) (Figure 2). The study sites were distributed over 4 continents including Europe (10 total, 3 Spain, 1 Netherlands, 2 Italy, 2 Czech Republic, 1 Denmark, 1 France) Asia (9 total, 4 Japan, 5 China), Oceania (8 total, all Australia) and North America (7 total, all USA). Only three of the five studies carried out in China were Chinese language publications [55], [61], [63].

### Summary estimates of sensitivity, specificity, LR and DOR

The pooled sensitivity and specificity estimates were 97.5% (95% confidence interval (CI): 96.8–98.5; I^2^ = 27.0%) and 95.8% (95% CI: 95.3–96.3; I^2^ = 91.6%), respectively (Figure S1). The results from the analysis of all the pooled manuscripts are also shown in Table 3.

**Table 3:** Summary results of the pooled and subanalysis by meta disc 1.4.

StatisticalIndex	Pooled	Sample Size^ϕ^		Instrument TypeΦ	
		Eligible^a^	Non-eligible	HR-1	Other§
**Sensitivity** **(%)**	97.7	99.3	96.6	95.1	98.7
	(96.8, 98.5)	(98.1, 99.8)	(94.9–97.8)	(92.0, 97.2)	(97.7, 99.3)
**Cochran's**	0.034	0.551	0.119	0.008	0.682
**Q (P value)**					
**I^2^**	27.0%	0.0%	19.9%	53.1%	0.0%
**Specificity**	95.8	93.4	96.2	99.5	95.4
**(%)**	(95.3, 96.3)	(91.7, 94.9)	(95.7, 96.7)	(98.6, 99.9)	(94.9, 95.9)
**Cochran's**	<0.0001	<0.0001	<0.0001	0.373	<0.0001
**Q (P value)**					
**I^2^**	91.6%	92.2%	91.6%	7.1%	93.2%
**LR +**	34.72	28.51	37.82	32.06	32.24
	(22.37, 53.90)	(9.80, 82. 92)	(22.61, 63.24)	(17.61, 58.37)	(19.88, 52.26)
**Cochran's**	<0.0001	<0.0001	<0.0001	0.539	<0.0001
**Q (P value)**					
**I^2^**	87.2%	93.2%	85.2%	0.0%	89.0%
**LR −**	0.07	0.02	0.10	0.10	0.06
	(0.05, 0.09)	(0.01, 0.04)	(0.08, 0.13)	(0.06, 0.17)	(0.04, 0.08)
**Cochran's**	0.132	0.95	0.815	0.078	0.780
**Q (P value)**					
**I^2^**	17.4%	0.0%	0.0%	36.5%	0.0%
**DOR**	711.75	2198.5	522.16	634.96	816.23
	(427.18,	(735.39,	(304.06,	(262.66,	(431.39,
	1185.9)	6572.6)	896.72)	1535.0)	1544.2)
**Cochran's**	0.031	0.31	0.102	0.829	0.004
**Q (P value)**					
**I^2^**	27.2%	14.4%	21.2%	0.0%	39.5%

§ : Other instruments included LightCycler480, Rotor-Gene6000, LightScanner96;

Φ : the sensitivity of the eligible and other instruments groups were significantly higher than the non-eligible and HR-1 groups, while the opposite relationship was observed for specificity (P<0.0001).

a : (>35 samples/amplicons with mutations and >35 samples/amplicons without mutations are needed to yield 95% confidence intervals whose lower bound is >90% sensitivity if the sensitivity is 100%).

### Meta-regression analysis

After single factor regression analysis, two variables were found to be explanatory: sample size and instrument type. Therefore, we developed a multivariable regression model using a backward stepwise algorithm to evaluate sample size and instrument type as variables. From this regression model, sample size was determined to be the most important characteristic (Table 2).

### Subgroup analysis

For the subgroup analysis of sample size and instrument type, the sensitivity of the eligible sample size subgroup was 99.3% (95% CI: 98.1, 99.8; I^2^ = 0.0%), non-eligible sample size subgroup was 96.6% (95% CI: 94.9, 97.8; I^2^ = 19.9%), other instruments subgroup was 98.7% (95% CI: 97.7, 99.3; I^2^ = 0.0%) and HR-1 instrument subgroup was 95.1% (95% CI: 92.0, 97.2; I^2^ = 53.1%) (Table 3). The sensitivity of the eligible sample size and other instruments subgroups were significantly higher than the non-eligible sample size and HR-1 instrument subgroups, respectively (P<0.0001, Figure S2 and Figure S3). The specificity of the eligible sample size subgroup was 93.4% (95% CI: 91.7, 94.9; I^2^ = 92.2%), non-eligible sample size subgroup was 96.2 (95% CI: 95.7, 96.7; I^2^ = 96.2%), other instruments subgroup was 95.4% (95% CI: 94.9, 95.9; I^2^ = 93.2%) and HR-1 instrument subgroup was 99.5% (95% CI: 98.6, 99.9; I^2^ = 7.1%). The specificity of the eligible sample size and other instruments subgroups were significantly lower than the non-eligible sample size and HR-1 instrument subgroups, respectively (P<0.0001, Figure S2 and Figure S3) but there were was no difference among other instruments within the other instruments subgroup (data not shown). No significant differences were detected in dye type and sample source subanalyses (data not shown).

The PRISMA 2009 checklist is provided as Checklist S1.

## Discussion

In this systematic review, we obtained summary estimates for the diagnostic accuracy of HRMA in the detection of disease-associated mutations in humans. HRMA was found to be a high sensitive modality when compared with DNA sequencing.

It has been previously shown that studies of diagnostic performance of modalities with methodological shortcomings may lead to overestimates of the accuracy of the diagnostic test [66]. In this study, meta-regression analysis was used to evaluate the effect of different study characteristics, such as sample size, instrument type and dye type, on the diagnostic performance of HRMA. The advantage of the regression analysis performed here is that the model accounts not only for the heterogeneity between studies from different threshold settings but also for the error of estimation of the sensitivity and specificity values in each study. This random model also accounts for the residual heterogeneity that may remain even after adjusting for individual study characteristics and HRMA technical conditions [67]. The results of the meta-regression analysis indicated sample size was the most significant characteristic influencing diagnostic accuracy.

Data from the subgroup analysis indicated differences for sample size. After studies were divided into eligible sample size and non-eligible sample size subgroups, the heterogeneity was significantly decreased. The eligible sample size subgroup of studies had significantly higher sensitivity and was less heterogeneous that the non-eligible sample size subgroup. These improvements may result from differences in the prevalence of samples/amplicons with mutations, as the number of mutations in the eligible sample size subgroup was significantly higher (550/1543, 35.6%), than the non-eligible sample size subgroup (697/6274, 11.1%). Therefore, the results showed that the number of samples with/without mutations in a study has an important influence on diagnostic accuracy [39].

Although the multivariable regression analysis presented here showed that the instrument type was not a significant characteristic, previous studies have shown that instrument type does affect the sensitivity and specificity of HRMA [11], [68]–[71]. The subgroup analysis of instrument type indicated some differences. For example, other instruments were more sensitive than the HR-1 instrument. This may be because the other instruments were some of the latest real-time thermal cyclers modified to incorporate HRMA, and yield high-resolution data quality by melting 18-times slower than the HR-1 instrument [25].

We found that HRMA was a highly sensitive method for mutation detection that yielded low negative LR without substantial heterogeneity. The sensitivity of all publications in the study, the eligible/non-eligible sample size subgroup and other instruments subgroups were 97.5%, 99.3%/96.6% and 98.7%, respectively and the negative LR were 0.07, 0.02/0.10 and 0.05, respectively. These results compare well with a recent compilation of 19 studies for constitutional variants that found an overall sensitivity of 99.3% (n = 5839) [72]. The high sensitivity of HRMA means that the technique can be considered as SnNOut (high sensitivity, negative, rules out) [73]–[75]. In this scenario, a negative HRMA test result rules out mutations. Therefore, when implemented correctly, the need for subsequent sequencing disappears for the pooled group (sensitivity 81.0%, 6322/7817, 32 false negatives), and the other instruments (sensitivity 82.2%, 5726/6967, 12 false negatives) and non-eligible sample size subgroups (sensitivity 85.6%, 5373/6274, 25 false negatives). These results are consistent with Provaznikova et al. [76] who reported avoiding unnecessary sequencing of more than 85% of the MYH9 gene. HRMA takes only a few minutes and costs only 11% of the cost of sequencing one exon [28], significantly reducing costs and saving time. However, in the eligible sample size subgroup, the reduction of sequencing is less (61.5%, 949/1543, 7 false negative) due to the greater number of mutations. Thus, the results showed HRMA is more suitable for screening for lower incidence mutations.

In general, as the sensitivity of diagnostic tests improves, the specificity decreases. Therefore, the specificity of the eligible sample size and other instruments subgroups was significantly lower than the non-eligible sample size and HR-1 subgroups. Specificity was homogeneous in the HR-1 instrument subgroup. This may be due to the fact that most of the samples were from only two research institutions (635/933 units of statistical analysis). However, the overall specificity of HRMA showed considerable heterogeneity between studies. This may be related to additional factors, such as the sequence length, GC content and sequence, that are properties of the individual sequences under study [77]–[79]. Other factors that are independent of the sequence, such as the presence of substances such as DMSO or betaine [80], [81], may also affect specificity. It is difficult to quantitatively analyse these factors.

In addition, we found that the sample source and dye used had no impact on HRMA accuracy. This is contrast to previous studies that found that the sample source and dye used affected HRMA accuracy [15], [20], [22], [41], [72]. The discrepancies between studies may result from differences in sample size, the focus of the researchers and/or the methods of statistical analysis. The continent of origin, design type and diagnostic accuracy also showed no significant effect in the meta-regression analysis.

In this study, amplicon length had some impact on the sensitivity and specificity of HRMA, as in previous reports [23], [82]. For example, for PCR products of less than 400 bp, sensitivity and specificity were 100%. While for PCR products 400–1000 bp long, the sensitivity was reduced to 96.1% and specificity to 99.4%. In this study, the majority of amplicon lengths were in the recommended amplicon length range (less than 300 bp) [23]. Therefore, the impact of amplicon length was not investigated further here. Many factors, including sequence-dependent and non-sequence dependent factors, affected HRMA accuracy. Therefore, standardization of DNA preparation, PCR and HRMA operating procedures are essential. Also, it remains necessary to subsequently sequence positive results from HRMA for confirmation. The current meta-analysis has some limitations in that the studies were heterogeneous and most studies used small sample sizes. In addition, the effect of language selection bias and literature type cannot be ignored as we only chose published articles in Chinese and English. In order to avoid this bias, the search should not be language limited and all literature types should be searched. However, most of the articles found on HRMA were published in English so the language bias was minimized. The selection bias was further minimized by maximizing the sensitivity of the search words, performing the search over a long search time and using a variety of databases/search engines including Medline, Embase, Cochrane Library, Medion, CBMdisc, Sciencedirect, SUMsearch, Google, Database for Chinese Journals of Technology (Chinese) and selected Journal Special Issues. In addition, the reference lists from articles obtained from the automated searches were checked manually.

Publication bias is a potential limitation of any systematic review. Smaller studies are associated with a greater diagnostic accuracy [83]. However, studies about publication bias focus mostly on randomized trials, and these types of studies are registered. The registration of studies for diagnostic studies is either limited or difficult to achieve. Due to the smaller sample sizes used for diagnostic studies, fewer studies were identified by the searches were for inclusion in this review. We examined publication bias by assessing whether the sample size of studies was associated with diagnostic accuracy, and found an association between sample size and HRMA diagnostic performance in the subgroup analysis. Therefore, assessment of the effect of sample size on HRMA accuracy was not ignored in further studies. In addition, there was no consistent relationship between language restriction and publication bias [83].

In conclusion, the sensitivity, simplicity, and low cost of HRMA make it the method of choice to screen patients for disease-associated variants, especially those diseases with lower incidence mutations. HRMA sensitivity is higher in the eligible sample size subgroup and is affected by instrument type but not by sample source or dye type. The DNA sequencing burden can be significantly reduced by the implementation of HRMA, but positive results still require sequencing for diagnostic confirmation. Further clinical studies of HRMA need to pay attention to the impact of sample size on diagnostic accuracy. However, as HRMA is still a relatively new technology, increases in accuracy can be expected as the diagnostic technology improves with time.

## Supporting Information

Figure S1 **Meta-analysis of studies evaluating HRMA sensitivity and specificity from pooled estimates.**
(TIF)

Figure S2 **Meta-analysis of studies evaluating HRMA sensitivity and specificity from the subanalysis of sample size.**
(TIF)

Figure S3 **Meta-analysis of studies evaluating HRMA sensitivity and specificity from the subanalysis of instrument type.**
(TIF)

Table S1 **Characteristics of the 34 studies included in the meta-analysis.**
(DOC)

Checklist S1 **PRISMA 2009 checklist.**
(DOC)
