# Supplementary figures and images for: Is High Resolution Melting Analysis (HRMA) Accurate for Detection of Human Disease-Associated Mutations? A Meta Analysis
Source: PLoS One. 2011 Dec 14;6(12):e28078. doi: 10.1371/journal.pone.0028078 (PMC3237421; doi:10.1371/journal.pone.0028078)

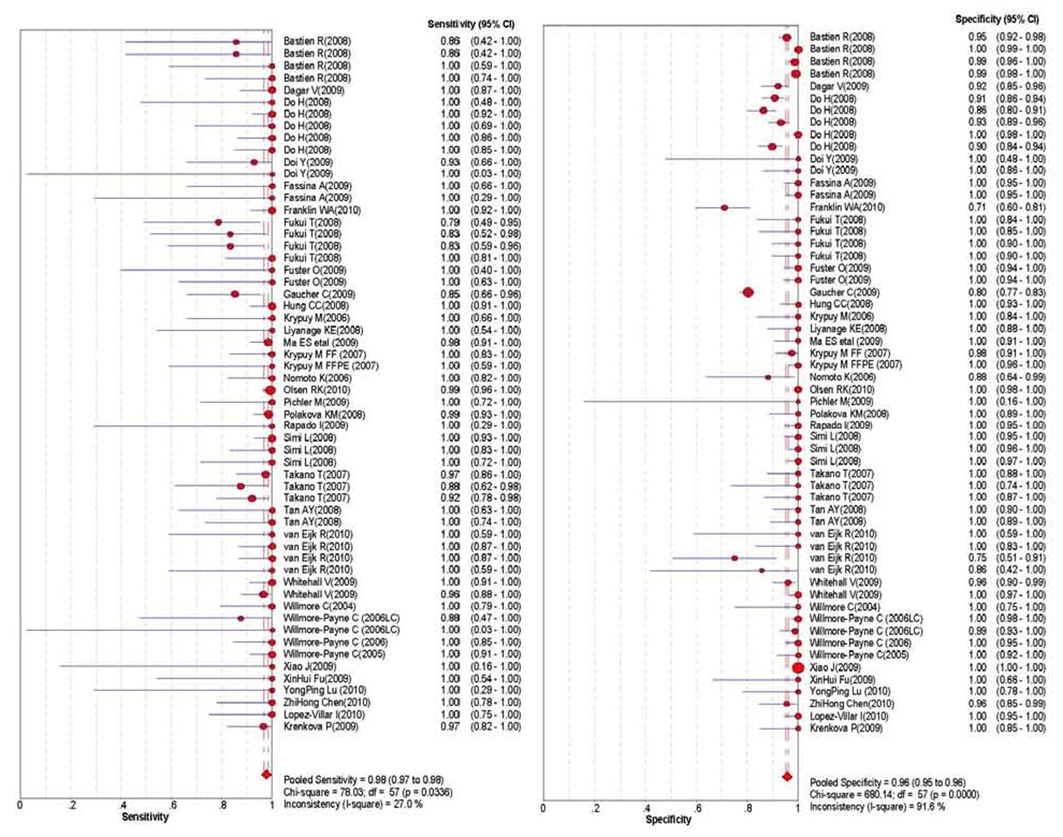

Supplement: Figure S1 — Meta-analysis of studies evaluating HRMA sensitivity and specificity from pooled estimates. (TIF) [file pone.0028078.s001.tif]

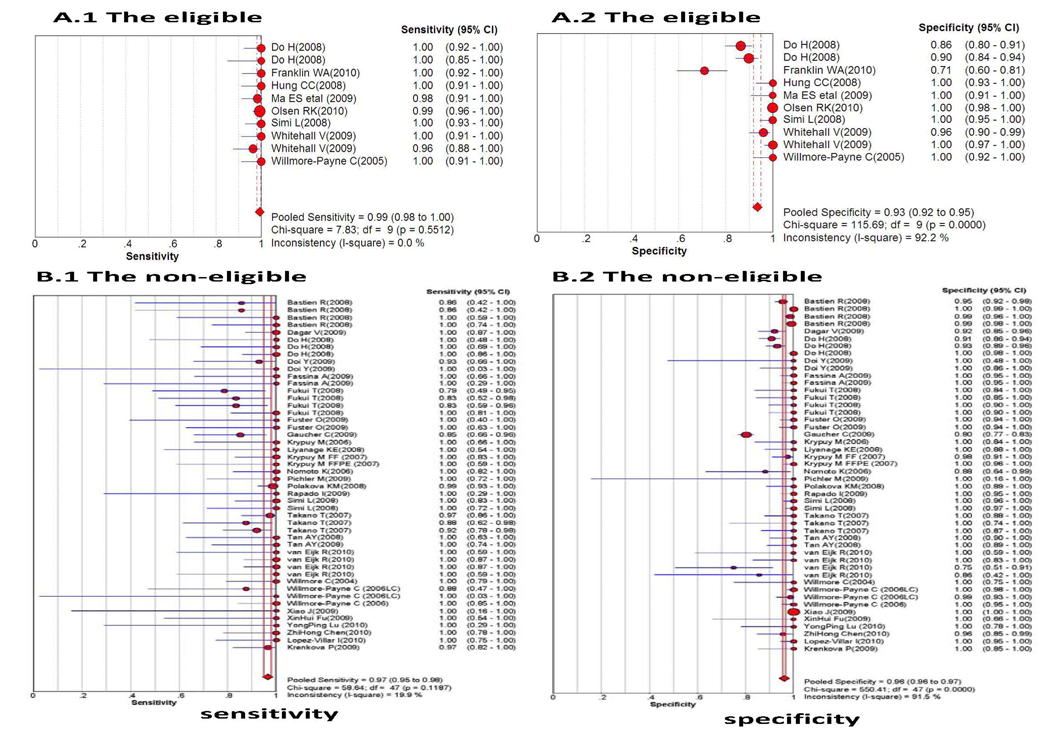

Supplement: Figure S2 — Meta-analysis of studies evaluating HRMA sensitivity and specificity from the subanalysis of sample size. (TIF) [file pone.0028078.s002.tif]

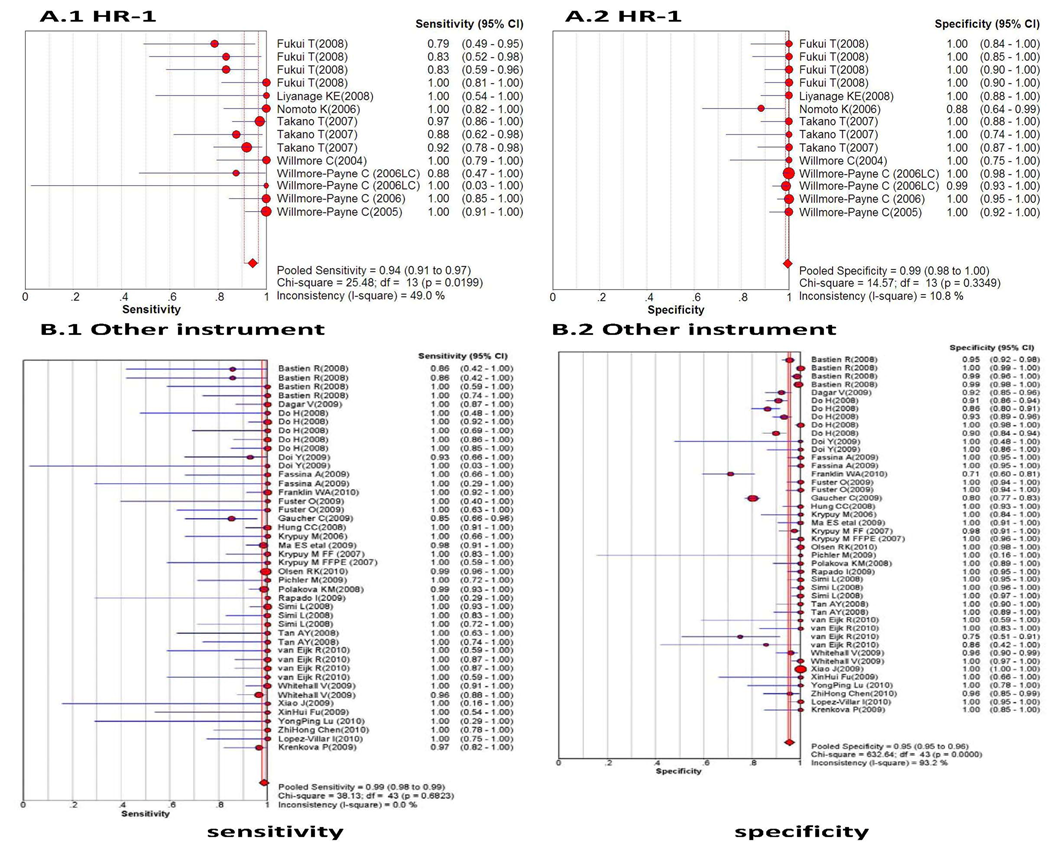

Supplement: Figure S3 — Meta-analysis of studies evaluating HRMA sensitivity and specificity from the subanalysis of instrument type. (TIF) [file pone.0028078.s003.tif]
