# Supplementary material for: Is High Resolution Melting Analysis (HRMA) Accurate for Detection of Human Disease-Associated Mutations? A Meta Analysis
Source: PLoS One. 2011 Dec 14;6(12):e28078. doi: 10.1371/journal.pone.0028078 (PMC3237421; doi:10.1371/journal.pone.0028078)
Supplement: Table S1 — Characteristics of the 34 studies included in the meta-analysis. (DOC) [file pone.0028078.s004.doc]

**Table S1**. Characteristics of the 34 studies included in the meta-analysis

| **Study ID** | **Disease/Specimen Source** | **Prevalence of**  **Samples with**  **Mutation^Ω^** | **Gene sequence/**  **Mutation-type Analyzed** | **Instrument/Dye/Level**  **of Analysis** | **Length**  **(bp)** | **Definition of Positive Result** | **TP** | **FP** | **FN** | **TN** |
| --- | --- | --- | --- | --- | --- | --- | --- | --- | --- | --- |
| **Bastien, R. (2008)^§^** | Breast cancer/FF | 2% (7/294) | TP53 exons/  2–11 (WGA) | LC480/LCG+  per amplicon | 80-150 | Mutation | 6 | 13 | 1 | 274 |
|  | Breast cancer/FFEP | 2% (7/294) | TP53 exons 2–11 |  |  |  | 6 | 0 | 1 | 287 |
|  | Breast cancer/FF | 2% (7/294) | TP53 exons 2–11 |  |  |  | 7 | 4 | 0 | 283 |
|  | Breast cancer/FF (23)**^ζ^** | 2% (12/483) | TP53 exons 2–11 |  |  |  | 12 | 4 | 0 | 467 |
| **Dagar, V. (2009)** | Rhabdoid tumors/FFEP | 19% (27/140) | SMARCB exons 1-9/23 Polymorphisms | RG/EG/per amplicon | 195-284 | Mutation/  Polymorphism | 27 | 9 | 0 | 104 |
| **Do, H. (2008)** | NSCLC/FFPE | 3% (5/200)  23% (46/200)  5% (10/200)  12% (23/200) | EGFR-18 | (RG)S9/per amplicon | 121-250 | Mutation | 5 | 18 | 0 | 177 |
|  | NSCLC/FFPE |  | EGFR-19 |  |  |  | 46 | 21 | 0 | 133 |
|  | NSCLC/FFPE |  | EGFR-20 |  |  |  | 10 | 13 | 0 | 177 |
|  | NSCLC/FFPE |  | EGFR-21/9 Polymorphisms |  |  | Mutation/  Polymorphism | 23 | 18 | 0 | 159 |
|  | NSCLC/FFPE | 13% (25/200) | Kras exon 2 |  | 92 | Mutation | 25 | 0 | 0 | 175 |
| **Doi, Y. (2009)** | Ph(+) leukemias/leukocyte | 74% (14/19) | bcr-abl kinase  domains-cDNA | LC480/RL/  per sample | 220-241 | Mutation | 13 | 0 | 1 | 5 |
|  | Ph(-)leukemias/leukocyte | 4% (1/26) | bcr-abl kinase  domains-cDNA |  |  |  | 1 | 0 | 0 | 25 |
| **Fassina, A. (2009)** | NSCLC/cytologic slides | 12% (9/77) | Kras exon 2 | LC480/RL/  per sample | 172 | Mutation | 9 | 0 | 0 | 68 |
|  | NSCLC/cytologic slides | 4% (3/77) | EGFR-21 L858R  mutation/ -19 DEL  region/1Polymorphisms |  | 142-190 | Mutation/  Polymorphism | 3 | 0 | 0 | 74 |
| **Franklin, W.A. (2010)** | Colon cancer/FFPE | 36% (42/118) | KRAS-exon2 | LC480/RL/  per amplicon | unclear | Mutation | 42 | 22 | 0 | 54 |
| **Fukui, T. (2008)** | NSCLC/cytologic slides | 40% (14/35) | EGFR-21 L858R  mutation/ -19 DEL | (HR-1)/LCG1  per sample | 51-83 | Mutation | 11 | 0 | 3 | 21 |
|  | NSCLC/FFPE(biopsy) | 35% (12/34) |  |  |  |  | 10 | 0 | 2 | 22 |
|  | NSCLC/FFPE (small samples) | 35% (18/52) |  |  |  |  | 15 | 0 | 3 | 34 |
|  | NSCLC/FFPE (resected specimens) | 35% (18/52) |  |  |  |  | 18 | 0 | 0 | 34 |
| **Fuster, O. (2009)** | AML/bone marrow | 6% (4/69)  12%(8/69) | KIT exon 8 | LC480/RL/  per amplicon | 138-213 | Mutation | 4 | 0 | 0 | 65 |
|  |  |  | KIT exon 17 |  |  |  | 8 | 0 | 0 | 61 |
| **Gaucher, C. (2009)** | Hypophosphatemic rickets/blood | 4% (27/724) | PHEX -11 exons**^＆^** | LC480/RL/  per amplicon | 229-634 | Mutation | 23 | 138 | 4 | 559 |
| **Hung, C.C. (2008)** | Achondroplasia/blood | 44% (40/90) | FGFR3-G1138A  mutation | HR-1/EG/  per amplicon | 249 | Mutation | 40 | 0 | 0 | 50 |
| **Krenkova, P. (2009)** | Cystic fibrosis/blood | 54% (28/52) | CFTR exons4, 7, 10, 11,  14b, and 22 | RG/LCG+/per sample | 101-380 | Mutation | 28 | 1 | 0 | 23 |
| **Krypuy, M. (2006)** | NSCLC/FF | 30% (9/30) | KRAS exon2 | RG/S9/per sample | 92-189 | Mutation | 9 | 0 | 0 | 21 |
| **Krypuy, M. (2007)** | Ovarian tumor/FF | 20% (20/100) | TP53-5-8/2 undetermined | RG/S9/per amplicon | 200-293 | Mutation | 20 | 2 | 0 | 78 |
|  | Breast tumor/FFPE | 7% (7/20) | TP53-5-8/1 ndetermined |  |  |  | 7 | 0 | 0 | 93 |
| **Liyanage, K.E. (2008)** | FDB/blood | 17% (6/35) | APOB exon 26 and  exon 29 | HR-1/EG/  per sample | 156-365 | Mutation | 6 | 0 | 0 | 29 |
| **Lopez-Villar, I. (2010)** | CRC/blood | 16% (13/82) | MUTYH  exons 7, 12 and 13 | LC480/RL/  per sample | 186-296 | Mutation | 13 | 0 | 0 | 69 |
| **Ma, E.S. (2009)^ζ^** | CRC/FFPE | 62% (62/100) | KRAS exon2/4  Known mutant | LC480/RL/  per sample | 170 | Mutation | 61 | 0 | 1 | 38 |
| **Nomoto, K. (2006)** | NSCLC/cytologic slides | 58% (21/36) | EGFR-21 L858R mutation/ -19 DEL/1 undetermined | HR-1/LCG1/  per amplicon | 51-83 | Mutation | 19 | 0 | 2 | 15 |
| **Olsen, R.K. (2010)** | VLCADD /blood or fibroblasts | 44% (146/329) | ACADVL 20exons /11 Polymorphisms | LS/LCG+/  per amplicon | 146-295 | Mutation/  Polymorphism | 145 | 0 | 1 | 183 |
| **Pichler, M. (2009)** | CRC/FFEP | 85% (11/13) | BRAF V600E | LC480/RL/  per sample | 147-238 | Mutation | 11 | 0 | 0 | 2 |
| **Polakova, K. M. (2008)** | CML/ leukocytes | 70% (73/104) | BCR-ABL1-4 | RG/LCG1/per amplicon | 221-241 | Mutation | 72 | 0 | 1 | 31 |
|  |  |  |  |  |  |  |  |  |  |  |
| **Rapado, I. (2009)** | myeloproliferative neoplasms/ blood | 4% (3/70) | JAK2 Exon 12 | LC480/RL/  per sample | 280 | Mutation | 3 | 0 | 0 | 67 |
| **Simi, L. (2008)** | CRC/FF | 45% (50/116) | KRAS exon 2 | RG/S9/per amplicon | 167 | Mutation | 50 | 0 | 0 | 66 |
|  |  | 17% (20/116) | PIK3CA exons 9 and 20 |  | 131-135 |  | 20 | 0 | 0 | 96 |
|  |  | 9% (11/116) | BRAF exon 15 |  | 250 |  | 11 | 0 | 0 | 105 |
| **Takano, T. (2007)** | advanced NSCLC/ Methanol-fixed | 56% (37/66) | EGFR-21L858R mutation/ -19 DEL | HR-1/LCG1/  per sample | 51-83 | Mutation | 36 | 0 | 1 | 29 |
|  | advanced NSCLC/  cytologic slides. | 57% (16/28) |  |  |  |  | 14 | 0 | 2 | 12 |
|  | advanced NSCLC/FFPE | 59% (37/63) |  |  |  |  | 34 | 0 | 3 | 26 |
| **Tan, A.Y.(2008)** | NK-AML/ bone marrow or  blood | 18% (8/44) | FLT3-ITD | LC480/RL/  per amplicon | 186-326 | Mutation | 8 | 0 | 0 | 36 |
|  |  | 27% (12/44) | NPM1 |  |  |  | 12 | 0 | 0 | 32 |
| **van Eijk, R. (2010)** | CRC/FFPE | 57% (26/46)  57% (26/46)  50% (7/14)  50% (7/14) | KRAS exon 2-3 | LS96/S9/  per amplicon | 166 | Mutation | 26 | 5 | 0 | 15 |
|  |  |  | KRAS exons 2-3(WGA) |  |  |  | 26 | 0 | 0 | 20 |
|  | CRC/ FF |  | KRAS exons 2-3(WGA) |  |  |  | 7 | 0 | 0 | 7 |
|  |  |  | KRAS exons 2-3 |  |  |  | 7 | 1 | 0 | 6 |
| **Whitehall, V. (2009)^＃^** | CRC/FFPE | 35% (56/160) | KRAS exons 2  KRAS exons 2 | LC480/S9  RG /S9 per amplicon | 80-92 | Mutation | 54 | 0 | 2 | 104 |
|  | CRC/FF | 29% (40/140) |  |  |  |  | 40 | 4 | 0 | 96 |
| **Willmore, C. (2004)** | GIST/FFPE | 55% (16/29) | KIT exons 9, 11, 13, 17 | HR-1/LCG1/  per sample | 170-235 | Mutation | 16 | 0 | 0 | 13 |
| **Willmore-Payne, C. (2005)** | Malignant melanoma/  FFPE | 48% (43/90) | BRAF exons 11,15/7 undetermined | HR-1/LCG1/  per amplicon | 190-250 | Mutation | 40 | 4 | 3 | 43 |
| **Willmore-Payne, C. (2006 LC)** | Lung adenocarcinoma/ FFPE | 5% (8/156) | EGFR exons 18-21/1 undetermined/  1Polymorphism | HR-1/LCG1/  per amplicon | 186-259 | Mutation/  Polymorphism | 7 | 0 | 1 | 148 |
|  |  | 3% (1/78) | HER2 exons19-20/  1undetermined |  | 214-252 |  | 1 | 1 | 0 | 76 |
| **Willmore-Payne, C. (2006)** | Squamous cell carcinoma  (head and neck)/FFPE | 23% (22/96) | EGFR exons 18-21/18 Polymorphisms | HR-1/ LCG1/  per amplicon | 186-259 | Mutation/  Polymorphism | 22 | 0 | 0 | 74 |
|  |  | 0% (0/48) | HER2 exons19-20**^τ^** |  | 214-252 |  | 0 | 0 | 0 | 48 |
| **Xiao, J. (2009)** | Primary dystonia/blood | 0.16% (2/1264) | TOR1Aexon5 and  c.863G>A | LC480/RL/  per amplicon | 314 | Mutation | 2 | 0 | 0 | 1262 |
| **YongPing, Lu. (2010)** | Non-syndrome cleft palate/ FF | 17% (3/18) | TGFA Taq | LS96/LCG1/  per sample | 179 | Mutation | 3 | 0 | 0 | 15 |
| **XinHui, Fu.,(2009)** | CRC/FFPE | 40% (6/15) | KRAS exons 2 | RG/EG/  per sample | 202 | Mutation | 6 | 0 | 0 | 9 |
| **ZhiHong, Chen. (2010)** | CRC/FF | 25% (15/60) | KRAS exons 2 | LC480/RL/  per sample | 189 | Mutation | 15 | 2 | 0 | 43 |
| **Total (unit of statistics**) - | | - | - | - | - | - | 1215 | 280 | 32 | 6290 |

FN: false-negative; FP: false-positive; TN: true-negative; TP: true-positive; LC: lung cancer; WGA: whole genome amplified; Ins: instrument; HR-1 = HR-1, LC = LC480, RG = Rotorgene6000, LS96 = LightScanner96; Dye: EG = Eva Green, LCG1 = LCGreen 1, LCG+ = LCGreen Plus, RL = Resolight, S9 = Syto 9; FF: Fresh frozen tissue; FFPE: formalin-fixed and paraffin-embedded; CRC: colorectal cancer; NSCLC: non small cancer lung cancer; AML: acute myeloid leukemia; GIST: Gastrointestinal stromal tumors; FDB: Familial ligand-defective apolipoprotein B-100; VLCADD: Very-long-chain acyl-CoA dehydrogenase deficiency; CML: chronic myeloid leukemia; NK-AML: normal karyotype acute myeloid leukemia;**ζ**according to the result of sequencing of HRM product; **#**: according to the result of dideoxy sequencing; **&**: select 11exons from 22exons;**§**optimal grouping sensitivity was included;τthe data were not included in statistical analysis;**Ω**: samples/amplicons are some samples/amplicons detected by HRMA and sequencing.
